# A case of enterovirus D68-associated acute flaccid myelitis in an immunocompromised adult, Australia

**DOI:** 10.1128/asmcr.00074-25

**Published:** 2025-08-21

**Authors:** Nathaniel Lizak, Grace Butel-Simoes, Eike Steinig, Belinda Cruse, Edrich Rodrigues, Kirsty Buising, Joe Sasadeusz, Marcelina Krysiak, Bruce Thorley, Matthew Kaye, Leon Caly, Jean Moselen, Ivana Savic, Andrew W. Roberts, Katherine Bond, Chuan Kok Lim, Deborah A. Williamson, Prashanth Ramachandran

**Affiliations:** 1 Department of Neurology, Royal Melbourne Hospital 393822, Melbourne, Victoria, Australia; 2 Victorian Infectious Diseases Reference Laboratory, The Royal Melbourne Hospital at The Peter Doherty Institute for Infection and Immunity 534133 , Melbourne, Victoria, Australia; 3 Department of Infectious Diseases, The University of Melbourne at the Peter Doherty Institute for Infection and Immunity 534133 , Melbourne, Victoria, Australia; 4 National Enterovirus Reference Laboratory, Victoria Infectious Diseases Reference Laboratory, Melbourne, Victoria, Australia; 5 Clinical Haematology, Royal Melbourne Hospital and Peter MaCallum Cancer Centre 90134, Melbourne, Australia; 6 Department of Microbiology, Royal Melbourne Hospital 90134, Melbourne, Australia; Vanderbilt University Medical Center, Nashville, Tennessee, USA

**Keywords:** acute flaccid myelitis, EV-D68

## Abstract

**Background:**

Acute flaccid myelitis (AFM) is a predominantly pediatric, polio-like syndrome associated with enterovirus D68. AFM in adults is rare, reported predominantly in immunocompromised hosts.

**Case Summary:**

Here, we report detection of enterovirus D68 in an immunocompromised Australian adult, specifically the B3 subgroup.

**Conclusion:**

This strain is phylogenetically linked to the 2018 AFM outbreaks in the USA and to documented cases in 2022 in France.

## INTRODUCTION

Enterovirus D68 (EV-D68) is a non-polio enterovirus associated with respiratory illness and, more rarely, acute flaccid myelitis (AFM), a polio-like syndrome predominantly reported in children. Adult-onset AFM is exceedingly uncommon and poses diagnostic and therapeutic challenges. We report a case of EV-D68-associated AFM in an immunocompromised adult in Australia, highlighting the diagnostic complexity, the role of metagenomic sequencing, and considerations for investigational antiviral use in severe neurotropic enterovirus infections.

## CASE PRESENTATION

### Initial presentation

A 30-year-old male presented with a background history of childhood Diamond-Blackfan anemia, complicated by refractory cytopenias, myelodysplasia, and requiring treatment with allogeneic stem-cell transplant, complicated by chronic graft-versus-host disease managed with varying levels of long-term immunosuppression. At presentation, his immunosuppressant regimen consisted of mycophenolate mofetil 1.5 g twice a day, prednisolone 17.5 mg daily, and tacrolimus 1.5 mg daily. Due to chronic hypogammaglobulinemia, he was receiving monthly intravenous immunoglobulin (IVIG). In September 2022, he presented with 2 weeks of cough, sore throat, and holocephalic headache; following initial discharge, he re-presented with worsening headache and low-grade fevers. There was no personal or family travel history outside of Australia. Nasopharyngeal PCR on presentation was positive for both Influenza A and for rhinovirus/enterovirus. He was admitted and commenced on oseltamivir 75 mg twice a day for 5 days.

**Fig 1:**
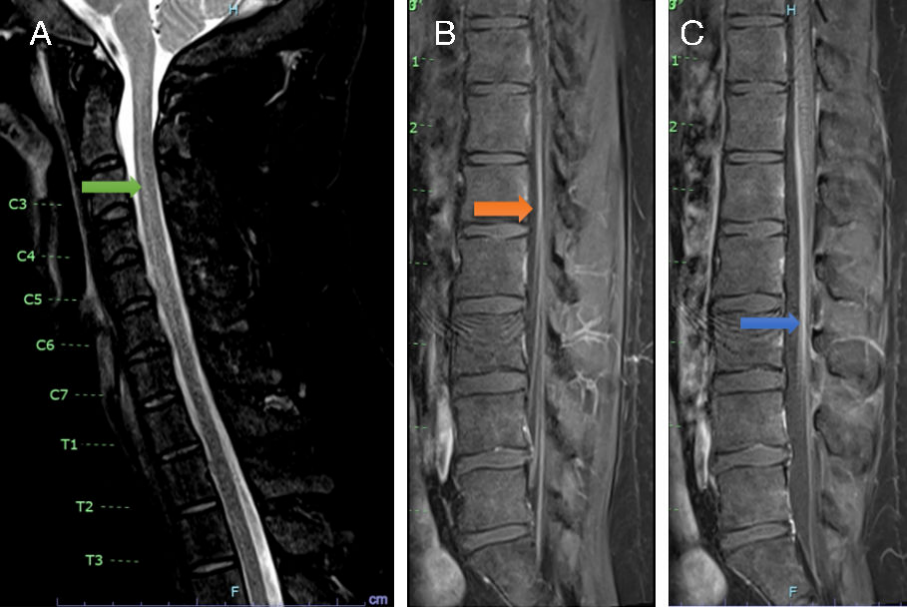
Spinal MRI. (**A**) MRI spine T2 sequences showing grey matter high T2 signal (green arrow). (**B and C**) Lumbosacral contrast-enhanced T1 MRI sequences showing smooth enhancement of the ventral surface of the cord (orange arrow) and of the cauda equina nerve roots (blue arrow).

Over the following 3 days, he continued to experience headaches and low-grade fevers and was also noted to develop new left arm pain and weakness and asymmetry of his lower face. On examination, he had lower-motor-neuron weakness of his left upper limb, which was flaccid and areflexic; in addition, he had weakness of the left trapezius and sternocleidomastoid, and a lower-motor-pattern left facial nerve palsy. There was subtle weakness of his right upper limb at the shoulder and triceps and hyporeflexia. He did not have meningism on examination. Cerebrospinal fluid (CSF) demonstrated a pleocytosis with 35 polymorphonuclear cells (ref <1 cells/μL), 14 lymphocytes (ref <5 cells/μL), and mildly elevated protein 0.57 g/L (ref <0.45 g/L) with normal glucose. The BioFire FilmArray Meningitis/Encephalitis (ME) Panel (testing for HSV1/HSV2/HHV6/VZV/enterovirus/CMV and bacterial/fungal pathogens) and herpes and enterovirus PCR testing were negative, as was cytology, flow cytometry, and extensive cultures for infective pathogens. Serum tests were largely unremarkable, except for hypogammaglobulinemia with serum IgG 2.10 g/L (ref 5.4–18.2g/L). Nerve conduction studies and electromyography demonstrated neurogenic changes with no active denervation and localized the lesion to either anterior horn or pre-ganglionic motor neuron pathways. MRI brain was non-contributory. Initial MRI spine (7 days post onset of neurological symptoms) had significant movement artifact and did not demonstrate a clear spinal cord lesion. Repeat MRI spine 10 days later demonstrated anterior gray matter high T2 signal in the cervical and upper thoracic cord, and smooth enhancement of the ventral surface of the distal cord from T11 to the conus involving the cauda equina nerve roots (Fig. 1).

**Fig 2:**
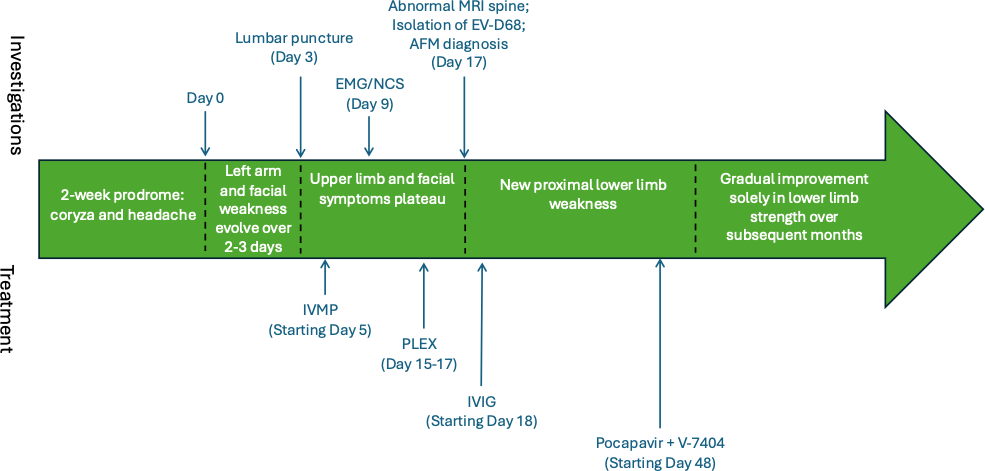
Summary of clinical course. EMG/NCS: electromyography/nerve conduction studies. AFM: acute flaccid myelitis. IVMP: intravenous methylprednisolone. PLEX: plasma exchange. IVIG: intravenous immunoglobulins.

Despite the patient’s investigative workup demonstrating central nervous system inflammation, it was unclear what the underlying etiology was but was considered to favor a post-infectious or autoimmune process, with other differentials, including lymphoproliferative or infective etiologies. Given the patient’s ongoing deterioration and no evidence of direct infection in his CSF, he was treated for an autoimmune etiology with 1 g intravenous methylprednisolone for 3 days, followed by two cycles of plasmapheresis. The clinical course is summarized in Fig. 2.

### Subsequent investigations

**Fig 3:**
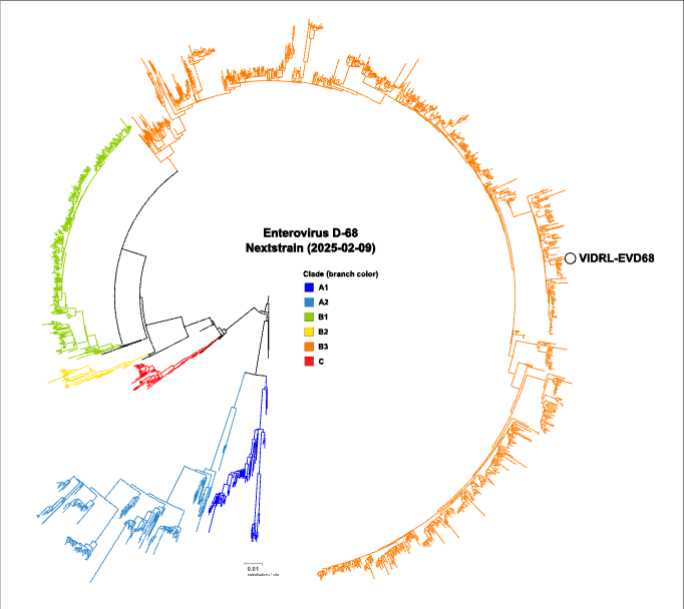
Maximum-likelihood phylogeny of the Nextstrain enterovirus D68 data set (2025-02-09), including the genome recovered in this study (VIDRL-EVD68). Branch colors indicate Nextstrain defined clades and sub-clades A–C. Strains in the immediate context of the VIDRL-EVD69 genome were collected in France (OP389245.1) and Italy (PQ414985.1–PQ414992.1, PQ415026.1–PQ415032.1, PQ415027.1, PQ415044.1–PQ415045.1) in the second half of 2022.

The admission influenza and enterovirus/rhinovirus-positive PCR was initially felt to represent a co-infection of flu and the common cold, but given the clinical presentation compatible with acute flaccid myelitis (AFM), ongoing workup for infectious etiologies was pursued. Further typing of the enterovirus detected in his nasopharyngeal swab was undertaken through VP1 amplification using Sanger sequencing. VP1 sequences were analyzed by comparison with a local database of prototype VP1 sequences and submission to NCBI NT with BLAST, version 2.13.0 (1). In addition, metagenomic next-generation sequencing (mNGS) and a targeted viral enrichment panel were performed on CSF and the nasopharyngeal swab. mNGS library preparation was performed based on previously described protocols (2). Data were processed through a custom clinical metagenomics pipeline (Cerebro v0.7.0 - https://github.com/esteinig/cerebro) with the pathogen detection and panviral enrichment workflows, as described previously (3). Enterovirus D68 (EV-D68) was detected with 3.48 rPM and 66% genome coverage with mNGS on the nasopharyngeal sample, aligning to NCBI: MK419070.1 (B3 subgroup). In the TWIST-enriched nasopharyngeal sample, 23,150 rPM aligned with 100% coverage and 510× depth. Subtyping confirmed EV-D68 B3 via multiple methods, including BLAST (99.96% coverage, 99.01% nucleotide identity to NCBI: OP389245.1) and phylogenetic analysis using the latest Nextstrain background data set (2025-02-09 [4–6]), with strains from France and Italy collected in 2022 forming the immediate clade context of the Australian genome (Fig. 3). Partial VP1 matched >99% nucleotide identity with French strains from 2021. Due to sampling bias in public databases, we avoided further conclusions about origin.

Reverse-transcription PCR, mNGS, and viral enrichment all failed to detect EV-D68 in CSF. Fecal enterovirus PCR was negative.

### Progress and management

With the confirmation of the B3 subgroup of EV-D68 from the nasopharyngeal swabs 17 days following the onset of neurological symptoms, it was felt that his clinical presentation was consistent with enterovirus myelitis with acute flaccid paralysis. He was commenced on high-dose IVIG 2 g/kg.

Despite IVIG, progressive neurological decline was observed, with unsteady gait. Examination demonstrated bilateral hip flexion weakness and bilateral Trendelenburg sign suggestive of proximal lower limb muscle involvement. Given the severity of his deficits, his immunocompromised state, and concerns for ongoing progression, he was considered for a trial of investigational oral antivirals pocapavir 1,600 mg daily and V-7404 2,000 mg twice a day for 14 days. There was a delay in accessing medications that were eventually started 31 days after the AFM diagnosis was confirmed. He had a prolonged admission under rehabilitation where he had improvement in his lower limb symptoms back to baseline but ongoing and persistent cranial nerve and upper limb weakness, with marked muscular atrophy.

## DISCUSSION

EV-D68-related AFM is a condition that is well studied in the pediatric population. Despite positive PCR from nasopharyngeal swabs during preceding respiratory infections, the causal link between the EV-D68 and acute flaccid myelitis (AFM) was initially questionable during the biennial AFM US outbreaks. This uncertainty stemmed from the absence of EV-D68 positive PCR results in the CSF of patients diagnosed with AFM (7). However, with time, there has been increasing evidence of direct viral invasion of the anterior horn cells of the spinal cord, leading to the characteristic clinic-radiological phenotype (8). While many enteroviral infections are known to be neurotropic, the phenotype of AFM has a stronger association with EV-D68 (9–11). Adult-onset EV-D68-associated AFM is relatively rare worldwide, especially in Australia, where even pediatric disease is seldom reported (12–14). Hypogammaglobulinemic patients are susceptible to enterovirus infections (15–17), though to our knowledge, this is the first case of EV-D68 associated AFM described in a hypogammaglobulinemic adult.

This case also demonstrates the complexity and diagnostic dilemma faced by clinicians. Testing for EV-D68 is extremely challenging, and this case required extensive specialized testing to achieve the diagnosis with delay, highlighting the unmet need for routinely available rapid tests for EV-D68. Having EV-D68 included into routine clinical respiratory panels may help clinicians be more aware of potential AFM cases leading to more specific and appropriate management. Considering the patient’s immunocompromised state and progressive neurological disability, we administered pocapavir and V-7404 treatments under compassionate use. It is unclear whether the improvement and stabilization of the lower limbs were due to antiviral therapy, the natural course of the disease, or supportive care.

Clinicians dealing with immunocompromised adults should be on alert for AFM as a potentially emerging complication of immunosuppressive treatments, including in non-endemic regions. Given the significant morbidity associated with neurotropic enterovirus infections, there is a definite need to enhance diagnostics, therapeutics, and markers predictive of therapeutic efficacy for these infections.

## Data Availability

Sequence data are available at NCBI/ENA/DDBJ under accession number PRJEB75233.
